# The Association of Targeted Gut Microbiota with Body Composition in Type 2 Diabetes Mellitus

**DOI:** 10.7150/ijms.51164

**Published:** 2021-01-01

**Authors:** Wei-Chun Hung, Wei-Wen Hung, Hui-Ju Tsai, Chen-Chia Chang, Yi-Wen Chiu, Shang-Jyh Hwang, Mei-Chuan Kuo, Szu-Chia Chen, Chia-Yen Dai, Yi-Chun Tsai

**Affiliations:** 1 Department of Microbiology and Immunology, College of Medicine, Kaohsiung Medical University, Kaohsiung, Taiwan.; 2 Division of Endocrinology and Metabolism, Department of Internal Medicine, Kaohsiung Medical University Hospital, Kaohsiung Medical University, Kaohsiung, Taiwan.; 3 Department of Family Medicine, Kaohsiung Municipal Ta-Tung Hospital, Kaohsiung Medical University, Kaohsiung, Taiwan.; 4 Division of Nephrology, Department of Internal Medicine, Kaohsiung Medical University Hospital, Kaohsiung Medical University, Kaohsiung, Taiwan.; 5 Faculty of Renal Care, College of Medicine, Kaohsiung Medical University, Kaohsiung, Taiwan.; 6 School of Medicine, College of Medicine, Kaohsiung Medical University, Kaohsiung, Taiwan.; 7 Department of Internal Medicine, Kaohsiung Municipal Hsiao-Kang Hospital, Kaohsiung Medical University, Kaohsiung, Taiwan.; 8 Division of Hepatobiliary, Department of Internal Medicine, Kaohsiung Medical University Hospital, Kaohsiung Medical University, Kaohsiung, Taiwan.; 9 Division of General Medicine, Department of Internal Medicine, Kaohsiung Medical University Hospital, Kaohsiung Medical University, Kaohsiung, Taiwan.; 10 Cohort Research Center, Kaohsiung Medical University, Kaohsiung, Taiwan.

**Keywords:** Type 2 diabetes mellitus, gut microbiota, lean tissue index, Firmicutes, Bacteroidetes

## Abstract

The association between body composition and gut microbiota in type 2 diabetes mellitus (DM) remains unknown. To elucidate the correlation of body composition and gut microbiota, we conducted a clinical study to enroll 179 patients with type 2 DM. Body composition of lean tissue index (LTI) and fat tissue index was measured by Body Composition Monitor. Eight pairs of 16S rRNA gene primers specific to Firmicutes, Bacteroidetes, the *Clostridium leptum* group, *Bacteroides*, *Bifidobacterium*, *Akkermansia muciniphila*, *Escherichia coli*, and *Faecalibacterium prausnitzii* were used to measure their abundance by quantitative polymerase chain reaction. The results showed that type 2 DM with higher abundance of phylum Firmicutes and a higher ratio of phyla Firmicutes to Bacteroidetes (phyla F/B ratio) had higher LTI. This significant correlation between phyla F/B ratio and LTI was especially evident in type 2 DM with high body mass index, and independent of glycemic control or dipeptidyl peptidase-4 inhibitor usage. In conclusion, our study demonstrated the positive association of LTI with the abundance of phylum Firmicutes and the phyla F/B ratio in type 2 DM.

## Introduction

Type 2 diabetes mellitus (DM) is a major health issue worldwide. The prevalence of type 2 DM is rapidly increasing globally, and is predicted to reach 629 million among those aged 20-79 years by 2045 1. Increasing evidence has demonstrated that disruption of gut microbiota greatly contributes to the development of type 2 DM 2,3.

Gut microbiota, trillions of microorganisms dwelling in the human gut, and weighing about 1.5 kg, has been regarded as a microbial organ with physical functions and a salient contributor to human health and disease 4. Dysbiosis, which has been demonstrated to provoke compositional changes of gut microbiota, alter the permeability of the intestinal barrier, and enhance metabolic endotoxin secretion, has been proved to contribute to many diseases, including obesity and type 2 DM 2,3. Phyla Firmicutes and Bacteroidetes, together accounting for more than 80% of total gut microbiota, are subdivided into more than 100 bacterial species 5. Although the importance of the ratio of phyla Firmicutes to Bacteroidetes (phyla F/B ratio) is still inconclusive 6, the study by Larsen et al. using real-time quantitative polymerase chain reaction (qPCR) reported that the proportion of Firmicutes is significantly decreased in patients with type 2 DM compared to normal individuals, and the phyla F/B ratios is negatively and significantly correlated with plasma glucose concentration 7.

Previous studies demonstrated that butyrate, which is digested from dietary fibers by certain bacterial species, maintains a tight junction and intestinal barrier function to reduce inflammation and improves insulin sensitivity 8-10. *Faecalibacterium prausnitzii*, which belongs to the *Clostridium leptum* group and also the Firmicutes phylum, makes a significant contribution to butyrate production 11. In patients with type 2 DM, the abundance of *F. prausnitzii* is decreased 12-14. Consequently, *Bifidobacterium* species, which display metabolic cross-feeding with butyrate-producing bacteria, are proportionally decreased in patients with type 2 DM 15. Recently, accumulating evidence shows *Akkermansia muciniphila* may improve glucose intolerance and adipose tissue inflammation, thus playing a role prior to the onset of type 2 DM 16,17. The gut microbiota also disrupts glucose metabolism and energy homeostasis. The phylum Proteobacteria produces lipopolysaccharide (LPS), which results in low-grade inflammation and increases intestinal permeability, leading to decreased insulin sensitivity 18,19. Increased abundance of *Escherichia coli* belonging to the Proteobacteria phylum has been reported in Chinese patients with type 2 DM 13.

Body composition, including muscle tissue, fat tissue, and the pattern of their distribution in the body, can reflect the health status and serve as the cause or consequence of complications in patients with type 2 DM 20,21. Previous study demonstrated that obese individuals with type 2 DM have different gut microbiome composition compared with those without type 2 DM 22. However, to our best knowledge, there is no reported study addressing the association of gut microbiota and body composition in patients with type 2 DM. Therefore, the aim of this study was to investigate the relationship between body composition and eight taxonomic units of which abundances have been reported to be related to type 2 DM, including the phyla Firmicutes and Bacteroidetes, the *C. leptum* group, genera *Bacteroides*, *Bifidobacterium*, *A. muciniphila*, *E. coli*, and *F. prausnitzii*, in patients with type 2 DM.

## Materials and Methods

### Study participants

This observational study was conducted in the outpatient department of a tertiary hospital in southern Taiwan from October 2016 to August 2017. Patients with antibiotic use in less than one month prior to enrollment, or with inflammatory bowel disease or with surgery of the gastrointestinal tract, or with currently diagnosed cancer undergoing chemotherapy in the previous year were excluded. Finally, we included 179 patients with type 2 DM in this study. The study protocol was approved by the Institutional Review Board of Kaohsiung Medical University Hospital (KMUHIRB-G(II)-20160021). Informed consent was obtained in written form from all of the patients, and all clinical investigations were conducted according to the principles expressed in the Declaration of Helsinki.

### Sample and clinical data collection

Diabetes was defined using blood glucose values based on American Diabetes Association criteria, a history of diabetes, or the use of anti-diabetic agents 23. Demographic statistics, including history of cigarette smoking and alcohol drinking, and clinical data were obtained from interviews with the patients and medical records at enrollment. Hypertension was defined as a history of hypertension or the use of antihypertensive drugs. Information on the use of medications, including anti-diabetic agents, statins, and anti-hypertensive agents, was obtained at enrollment from medical records. Body mass index (BMI) was calculated as body weight in kilograms divided by body height in squared meters. All study participants were enrolled in the diabetic education program and the principles of diet therapy to diabetes were delivered on individual basis. We recorded usual diet habits in these patients by a simple questionnaire as listed in **Table 1.** The patients were asked to fast for at least 12 hours before blood was taken for biochemical study.

### Measurement of body composition

Body composition was measured once using a bioimpedance spectroscopy method, Body Composition Monitor (BCM, Fresenius Medical Care, Germany) at the same day of collecting blood samples. The BCM measures the impedance spectroscopy at 50 different frequencies from 5 kHz to 1 MHz, and has been validated against gold-standard methods in the general population 24,25. Patients had been in the recumbent position for at least 5 minutes, and then electrodes were attached to one hand and one foot on the ipsilateral side. The measurement results were optimized, and raw data was adapted to the model function. The quality of the raw data was displayed as the value Q, with values near 100 representing high data quality and near 0 representing low data quality. Only the parameters for which the quality of the measurement was 95% or more were included in the analysis. The BCM can distinguish muscle mass and fat mass from pathologic fluid retention in the body based on the difference of impedance in each tissue through a new 3-component tissue-based model 26 and provide information including normohydrated lean tissue, normohydrated adipose tissue, and excess fluid mass 27. Normohydrated lean tissue and normohydrated adipose tissue were presented as lean tissue index (LTI, lean tissue mass/height^2^) and fat tissue index (FTI, adipose tissue mass/height^2^), respectively 26. The normal ranges of LTI and FTI are 12-17 kg/m^2^ and 4.25-10.5 kg/m^2^ respectively. The output parameters fit into the same reference ranges set by Fresenius Medical Care (Germany) 26,28.

### Stool sample collection and microbial DNA extraction

Fecal samples were collected by patients at home, immediately frozen in the household freezer, and brought to the hospital within 12 hours. The time of fecal sample collection was either the evening one day before or the morning on the same day of obtaining biochemical data. Then, fecal samples were transferred to the laboratory and stored at -80 °C for up to three days before processing. Bacterial DNA was extracted using the Stool DNA Extraction kit (Topgen Biotechnology Co., Ltd, Kaohsiung, Taiwan). In brief, the fecal samples were weighed to 50-100 mg and were supplemented with a preceding bead beating (45 seconds; speed: 3450 oscillations/min). The subsequent steps of DNA extraction were performed according to the manufacturer's protocol. DNA concentration and quality was assessed by Colibri Microvolume spectrophotometer (Titertek Berthold, Pforzheim, Germany). Extracted DNA samples were immediately stored at -20 °C before use.

### Real-time quantitative polymerase chain reaction (qPCR)

Real-time qPCR was utilized to measure bacterial 16S rRNA gene copies in feces in the StepOnePlus Real-Time PCR system (Thermo Fisher Scientific, Waltham, MA, USA) as in a previous study 29. Eight pairs of 16S rRNA gene primers specific to Firmicutes, Bacteroidetes, the *C. leptum* group, *Bacteroides*, *Bifidobacterium*, *A. muciniphila*, *E. coli*, and *F. prausnitzii* are listed in **Supplementary Table 1.** Standard curves were constructed with a 10-fold dilution series of the 16S rRNA gene fragment amplified from the reference strains that was cloned into a T&A^TM^ Cloning Vector (Yeastern Biotech, Co., Ltd, Taipei, Taiwan). Each reaction mixture with a total volume of 10 μl was composed of 0.25 μl of each 10 μM primer, 5 μl of AceQ qPCR SYBR Green Master Mix (Vazyme Biotech Co., Piscataway, NJ, USA), 1 μl of sample DNA, and 3.5 μl sterilized ultra-pure water. Real-time PCR was carried out by the following cycle conditions: an initial holding at 95 °C for 30 s, followed by 40 cycles of denaturation at 95 °C for 3 s, then annealing/elongation at 60 °C for 40 s. Melting curve analysis was performed after amplification to determine the specificity. Quantitation of the eight taxonomic units was evaluated as the copy numbers of the 16S rRNA genes/gram of feces weight. All qPCR tests were performed in duplicate, and the presented data are the mean values of duplicate qPCR analysis.

### Statistical Analysis

Continuous variables are presented with mean ± SD or median (25^th^, 75^th^ percentile), and those with skewed distribution were log-transformed to approximate a normal distribution. Categorical variables are presented as percentages. The significance of differences in continuous variables between groups was analyzed using the Kruskal-Wallis H test. The chi-squared test was utilized to test differences in the distribution of categorical variables. Linear regression was used to evaluate the determinants of body composition in the patients with type 2 DM. All the variables in **Table 1** tested by univariate analysis and those variables with *p* value less than 0.05, age, and sex were selected in a multivariate linear regression analysis. Statistical analyses were conducted using SPSS version 18.0 for Windows (SPSS Inc., Chicago, Illinois) and graphs were drawn using Graph Pad Prism 5.0 (GraphPad Software Inc., San Diego CA, USA). Statistical significance was set at a two-sided *p* value of <0.05.

## Results

### Characteristics of the entire cohort

**Table 1** reveals the clinical characteristics, medication records, microbial abundance, and laboratory parameters of the entire cohort. Of the 179 subjects, the mean age was 63.2 ± 10.2 years, 55.3% were male, and the median of diabetic duration was 10.0 (5.0, 15.0) years. The prevalence of hypertension and hyperlipidemia was 66.5% and 82.7%, respectively. The means of BMI, LTI, and FTI were 26.3 ± 4.0, 11.8 ± 2.3, and 14.1 ± 4.4 kg/m^2^, respectively. The median of HbA1c was 6.9% among study subjects.

### Gut microbiota and LTI in the patients with type 2 DM

The medium of bacterial concentrations (estimated as copy numbers of 16S rDNA per gram of feces) are listed in Table 1. The positive associations of LTI with the phylum Firmicutes (Spearman's rho = 0.213, *p*-value = 0.004) and phyla F/B ratio (Spearman's rho = 0.239, *p*-value = 0.001) were shown in the patients with type 2 DM (**Table 2**). Because of abnormal distribution of gut microbiota amount, we stratified type 2 diabetic patients according to tertiles of LTI (10.6 and 12.7 kg/m^2^) to precisely analyze the distribution of gut microbiota among these patients with different level of LTI (**Table 3**). The stepwise increase in the abundance of the phylum Firmicutes and the F/B ratio was found from tertile 1 to tertile 3. There was a significant difference in the abundance of *Bacteroides* and *A. muciniphila* among LTI tertiles. However, no stepwise increase in the abundance of *Bacteroides* and *A. muciniphila* was shown from tertile 1 to tertile 3 in the patients with type 2 DM. There was no difference in the abundance of Bacteroidetes, *Bifidobacterium*, *E. coli*, or *F. prausnitzii* in the patients with different LTIs. We also stratified these patients based on LTI of median to evaluate the distribution of gut microbiota and the results were similar with our current results (**Supplementary Table 2**).

We performed linear regression analysis to analyze the determinants of LTI (**Table 4**). In univariate analysis, male, BMI, serum hemoglobin, creatinine and log-formed albumin levels, phylum Firmicutes, and phyla F/B ratio were significantly and positively associated with LTI in the patients with type 2 DM. Age and log-formed serum phosphate level were negatively correlated with LTI. Further multivariate analysis revealed that the patients with higher abundance of phylum Firmicutes (β = 0.65, 95% confidence index (CI) = 0.06-1.23) and higher phyla F/B ratio (β= 0.48, 95% CI = 0.02-0.94) had higher LTI. However, there was no significant correlation between gut microbiota and FTI in the patients with type 2 DM (**Table 2 and Supplementary Table 3**).

### Gut microbiota and LTI of the patients with type 2 DM in different subgroups

**Figure 1:**
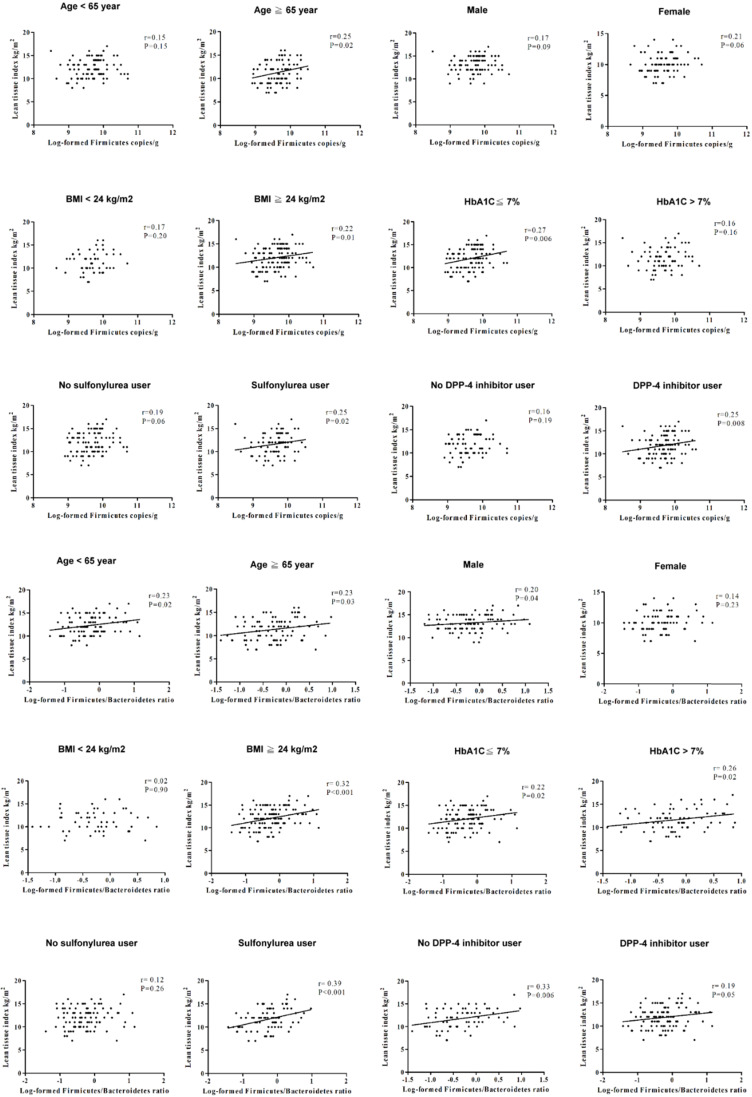
The correlation between lean tissue index and the phylum Firmicutes or the phyla F/B ratio in different subgroups.

In order to investigate the effect of age, sex, glycemic control, and anti-diabetic agents usage on LTI, we also stratified the patients with type 2 DM by age (65 years-old as cut-off value for old age), sex, HbA1c (7% as an index for optimal glycemic control), and anti-diabetic agents (**Figure 1**), and the results revealed a positive association of LTI with the phylum Firmicutes in the patients with age ≥65 years-old, HbA1c ≤7%, or using sulfonylurea and dipeptidyl peptidase-4 (DPP4) inhibitors. The correlation between LTI with the phyla F/B ratio was found in the patients with male gender, or using sulfonylurea. The correlation between LTI with the phyla F/B ratio was independent of age, HbA1c level, and using DPP4 inhibitors in the patients with type 2 DM. We also stratified the patients with type 2 DM by BMI (24 kg/m^2^ as cut-off value for definition of overweight in Taiwanese population 30 (**Figure 1**), and found a positive correlation between LTI and the phylum Firmicutes and the phyla F/B ratio in the patients with BMI ≥24 kg/m^2^, not in those with BMI <24 kg/m^2^.

## Discussion

This is the first study to evaluate the association of the gut microbiota with body composition in the patients with type 2 DM. We found that the phylum Firmicutes and the phyla F/B ratio were significantly and positively correlated with LTI. The patients with higher abundance of Firmicutes and a higher phyla F/B ratio were more likely to have more lean mass after adjusting for a number of variables of body composition, such as age, sex, and BMI. This significant correlation between phyla F/B ratio and LTI was especially evident in the patients with type 2 DM who had high BMI, and was independent of glycemic control or DPP4 inhibitor usage.

Accumulating evidence reveals that the phyla F/B ratio is higher in obese individuals than lean individuals, and the phyla F/B ratio is positively correlated with BMI 31. BMI has been widely used as an indicator of health. However, BMI cannot provide accurate information about the distribution of body composition. Body composition, in terms of lean tissue and fat tissue, can provide a more accurate reflection of physical function and nutrition status compared with BMI 32. Lean mass has been presented as a nutritional marker, and is correlated with increased risks for osteoporosis, physical disability, functional impairment, hospitalization, and even mortality in the general population 33-35. Loss of lean tissue may result in insulin resistance, inflammation, and micro- and macrovascular complications in patients with type 2 DM 36,37. The patients with type 2 DM usually have a lower phyla F/B ratio than normal individuals 7. The current results demonstrated the positive association of LTI with Firmicutes and the phyla F/B ratio in the patients with type 2 DM. The possible explanations are that Firmicutes may utilize energy sources more effectively than Bacteroidetes, and Firmicutes is correlated with the nutrient transporter, thus enhancing the lean tissue amount 18. Furthermore, variation of LTI has been associated with all-cause mortality or cardiovascular events 38. Based on the significant correlation between microbiota and LTI, microbiota might be the potential biomarkers to evaluate outcomes in clinical patients. Further study is needed to examine whether type 2 diabetic patients with higher abundance of *Firmicutes* or higher phyla F/B ratio have better clinical outcomes than those with lower abundance of *Firmicutes* or lower phyla F/B ratio.

Grosicki et al. suggest that the gut microbiota plays a principle role in the metabolism of lean tissue 18. Previous study demonstrated that butyrate-producing bacteria (the *C. leptum* group and *F. prausnitzii* in this study) may reduce inflammation, leading to enhanced muscle function 8,9. *Bifidobacterium* may affect gut-muscle communication and modulate muscle size, and supplementation of *Bifidobacterium* probably reduces muscle wasting 18. The phylum Proteobacteria produces LPS and increases intestinal permeability, triggering systemic inflammation and muscle maladaptation 18. However, our results did not find an association of LTI with the *C. leptum* group, *F. prausnitzii, Bifidobacterium*, or* E.coli* in the patients with type 2 DM. This inconsistent finding may relate to differences in study population, race, and diet habit. It has been reported that supplementation with *A. muciniphila* slightly decreased fat mass in overweight or obese individuals 16. Our results showed significant difference but no stepwise increase in the abundance of *A. muciniphila* (**Table 3**), which might be due to the wide distribution range of the single microbial species seen in our study with small sample size.

The gut microbiota regulates the amount, distribution, and storage of fat tissue in mice 39. However, our results did not reveal a significant correlation between fat tissue and gut microbiota in the patients with type 2 DM. The relatively small number of the patients with type 2 DM may underestimate the effect of gut microbiota on the alteration of fat tissue. Furthermore, this study only measured eight targeted gut microbiota, and other bacteria related to fat tissue might not be examined. In addition, muscle wasting is probably one of the reasons why microbiota was correlated with LTI, and not with FTI, in the patients with type 2 DM. In accordance with the measured LTI compared with the age- and gender-normalized LTI value, an LTI less than 10% of the normal value indicates muscle wasting 40. These patients were under stable clinical condition and had high serum albumin level (4.7 ± 0.2 g/dl), as a nutrition marker, meaning that they might not have presented with the real status of muscle wasting. It is necessary to examine the interactional effect of muscle wasting on the correlation between microbiota and body composition in the future.

Overweightness and obesity are common in type 2 DM 41. The composition of gut microbiota may be altered in overweight or obese patients with type 2 DM. We stratified the patients with type 2 DM by BMI of 24 kg/m^2^, as the cut-off value for the definition of being overweight in the Taiwanese population 30, to analyze the correlation between LTI and gut microbiota. We found a positive association of LTI with both the abundance of Firmicutes and the F/B ratio in the patients with BMI ≥24 kg/m^2^, but not in those with BMI <24 kg/m^2^. The abundance of Firmicutes and the F/B ratio may influence lean tissue mass in the patients with type 2 DM, especially those with high BMI.

Type 2 DM is associated with an altered amount and distribution of lean tissue and fat tissue 35. Wierzbicka et al. demonstrated that the patients with type 1 DM having lower HbA1c levels had elevated lean mass 42. However, there was no correlation between body composition variables and HbA1c in type 2 DM 43,44. The relationship between HbA1c and body composition is controversial in patients with DM. In addition, plasma DPP4 activity is reported to be positively correlated with lean mass and central adiposity, and negatively with general adiposity 45. Sulfonylurea has the well-known side effects of weight gain 46, which might lead to change body composition. Therefore, we further divided the patients with type 2 DM into HbA1c ≤7% or >7%, and users or non-users of DPP4 inhibitors or sulfonylurea. The correlation between LTI and the phyla F/B ratio was independent of the HbA1c level and DPP4 inhibitor usage. This positive correlation was shown in sulfonylurea users of the patients with type 2 DM. The mechanism for sulfonylurea to affect body composition may be mediated by gut microbiota.

In our study, more than 80% of participants with type 2 DM were treated with metformin, and there was no significant difference of the abundance of eight bacteria between the patients with and without metformin usage (**Supplementary Table 4**). Thus, metformin is not a confounder in this study. Previous literature demonstrated that metformin would induce the variation of microbiome composition 47-51. The contradiction might be due to the experimental methods. Our study implemented qPCR to compare the eight targeted microbial species instead of analyzing the whole gut microbiome with 16S rRNA gene sequencing in many other studies.

Our study disclosed for the first time that associations might exist between gut microbiota and body composition in the patients with type 2 DM. However, three are some limitations in this study. Firstly, only eight targeted gut microbiota were measured by real-time qPCR instead of by 16S rRNA sequencing. Compared to 16S RNA sequencing, real-time qPCR is cost-effective and time-saving and provides the ability to measure the absolute quantity rather than the relative percentage. However, without analyzing gut microbiota with the gold-standard method of 16S rRNA sequencing, our results fail to present the whole microbiome signature pertaining to body composition. Further advanced study using 16S rRNA sequencing would be needed to reveal the whole profile of the gut microbiota associated with body composition. Moreover, the cross-sectional design of this study does not allow demonstration of the cause-effect relationship of gut microbiota and body composition, and might even lead to random results. Future longitudinal study of gut microbiota and body composition is needed and a separate cohort study should be conducted to confirm our novel finding. Finally, although we recorded usual diet habits, the data of detailed diet content (total energy, carbohydrate, protein, and fat intake) is lacking, and might underestimate the impact of diet on the correlation between microbiota and body composition.

In conclusion, this study demonstrated for the first time the relationship between gut microbiota and body composition in the patients with type 2 DM. The abundance of Firmicutes and the phyla F/B ratio were significantly associated with lean tissue. Further study will be conducted to analyze the interaction between gut microbiota and body composition in clinical outcomes of the patients with type 2 DM.

## Supplementary Material

Supplementary figures and tables.

## Figures and Tables

**Table 1:** The clinical characteristics of study subjects

	Entire cohort N = 179
Age, year	63.2 ± 10.2
Sex (male), %	55.3
Smoke, %	21.8
Alcohol, %	16.2
Cardiovascular disease, %	16.2
Hypertension, %	66.5
Hyperlipidemia, %	82.7
Gout, %	7.8
BMI, kg/m^2^	26.3 ± 4.0
Mean arterial pressure, mmHg	98.2 ± 11.6
Diabetic duration, years	10.0 (5.0,15.0)
LTI, kg/m^2^	11.8 ± 2.3
FTI, kg/m^2^	14.1 ± 4.4
**Diet habit, %**	
Protein more than fiber	13.1
Fiber more than protein	32.7
Fiber equal to protein	54.2
**Medications**	
Novonorm user, %	5.0
Sulfonylurea user, %	46.4
DPP4-inhibitor user, %	61.5
Metformin user, %	81.6
Insulin user, %	15.1
Statin user, %	49.7
**Microbiota**	
Firmicutes, copies × 10^9^/g	5.31 (2.38, 8.65)
Bacteroidetes, copies × 10^9^/g	10.16 (4.39, 19.94)
Firmicutes/Bacteroidetes	0.54 (0.24, 1.10)
*C. leptum* group, copies × 10^8^/g	7.69 (2.64, 13.37)
*Bacteroides*, copies × 10^9^/g	2.04 (0.94, 4.34)
*Bifidobacterium*, copies × 10^6^/g	1.77 (0.19, 10.65)
*A. muciniphila*, copies × 10^5^/g	0.15 (0.05, 175.52)
*E. coli*, copies × 10^8^/g	1.23 (0.34, 5.82)
*F. prausnitzii*, copies × 10^7^/g	10.69 (2.25, 31.40)
**Laboratory parameters**	
HbA1c, %	6.9 (6.5, 7.9)
Creatinine, mg/dl	0.9 (0.7,1.11)
Hemoglobin, g/dl	13.9 ± 7.5
Albumin, g/dl	4.6 (4.3, 4.7)
Calcium, mg/dl	9.3 ± 0.4
Phosphate, mg/dl	3.7 (3.3, 4.0)
Uric acid, mg/dl	5.9 ± 1.5
Cholesterol, mg/dl	165 (144, 191)
Triglyceride, mg/dl	117 (83, 180)

Abbreviations: BMI, body mass index; DPP4, dipeptidyl peptidase-4; FTI, fat tissue index; LTI, lean tissue index. Data are expressed as number (percentage) for categorical variables and mean ± SD or median (25th, 75th percentile) for continuous variables, as appropriate.

**Table 2:** The correlation among microbiome, lean tissue index, fat tissue index and body mass index in study subjects

		Firmicutes (copies/g)	Bacteroidetes (copies/g)	Phyla F/B ratio	*C. leptum* group (copies/g)	*Bacteroides* (copies/g)	*Bifidobacterium* (copies/g)	*A. muciniphila* (copies/g)	*E. coli* (copies/g)	*F. prausnitzii* (copies/g)
LTI (Kg/m^2^)	Spearman's rho	0.213	-0.077	0.239	0.018	-0.068	0.084	-0.118	0.052	-0.038
	*P* value	0.004	0.307	0.001	0.808	0.367	0.262	0.116	0.49	0.618
FTI (Kg/m^2^)	Spearman's rho	-0.127	-0.043	-0.067	-0.047	-0.038	-0.112	-0.026	0.018	-0.087
	*P* value	0.09	0.568	0.373	0.535	0.616	0.137	0.727	0.809	0.246
BMI (Kg/m^2^)	Spearman's rho	-0.021	-0.075	0.042	-0.028	-0.085	-0.08	-0.063	0.063	-0.123
	*P* value	0.784	0.318	0.579	0.714	0.259	0.285	0.405	0.401	0.1

Abbreviations: BMI, body mass index; FTI, fat tissue index; LTI, lean tissue index.

**Table 3:** The microbiota of the study subjects stratified by lean tissue index

Lean tissue index	Tertile 1 (N=60)	Tertile 2 (N=60)	Tertile 3 (N=59)	*P* value
Firmicutes, copies × 10^9^/g	3.82 (2.21, 5.97)	5.91 (2.77,10.30)	5.91 (2.82,9.67)	0.03
Bacteroidetes, copies × 10^9^/g	10.22 (4.60,24.73)	12.81 (6.58,23.54)	8.24 (3.78,14.92)	0.06
Phyla F/B ratio	0.38 (0.15,0.75)	0.51 (0.28,0.92)	0.69 (0.37,1.85)	0.004
*C. leptum* group copies × 10^8^/g	6.87 (2.20,13.30)	8.64 (4.33,14.92)	6.05 (2.26,13.52)	0.30
*Bacteroides*, copies × 10^9^/g	2.04 (0.95,5.39)	2.72 (1.36,4.71)	1.47 (0.55,2.82)	0.03
*Bifidobacterium*, copies × 10^6^/g	1.76 (0.49,11.73)	1.87 (0.13,10.76)	2.05 (0.58, 10.64)	0.67
*A. muciniphila*, copies × 10^5^/g	0.17 (0.05,459.78)	0.38 (0.07,657.71)	0.09 (0.03,9.92)	0.02
*E. coli*, copies × 10^8^/g	1.04 (0.31,6.37)	2.60 (0.55,7.81)	1.16 (0.27,4.29)	0.22
*F. prausnitzii*, copies × 10^7^/g	8.89 (1.70,25.80)	15.98 (3.20,36.19)	8.43 (2.37,31.45)	0.45

**Table 4:** The determinants of lean tissue index in study subjects

	Univariate		Multivariate (stepwise)		Multivariate (stepwise)	
	β (95%Cl)	*P* value	β (95%Cl)	*P* value	β (95%Cl)	*P* value
**Clinical characteristics**						
Age, year	-0.06 (-0.10, -0.03)	<0.001	-0.06 (-0.08, -0.03)	<0.001	-0.06 (-0.08, -0.03)	<0.001
Sex (male), %	3.08 (2.58, 3.58)	<0.001	2.95 (2.47, 3.43)	<0.001	2.98 (2.51, 3.45)	<0.001
Heart disease, %	0.19 (-0.73, 1.10)	0.69	--	--	--	--
Body mass index, kg/m^2^	0.12 (0.04, 0.20)	0.005	--	--	--	--
Mean arterial pressure, mmHg	0.00 (-0.03, 0.03)	0.82	--	--	--	--
**Diet habit, %**						
Protein more than fiber	-0.71 (-1.94, 0.53)	0.26				
Fiber more than protein	0.56 (-0.33, 1.45)	0.21				
**Microbiome (log-formed)**						
Firmicutes, copies*10^9^/g	1.01 (0.20, 1.82)	0.02	0.65 (0.06, 1.23)	0.03	--	--
Bacteroidetes, copies*10^9^/g	-0.08 (-0.59, 0.42)	0.75	--	--	--	--
Firmicutes/Bacteroidetes	1.06 (0.43, 1.70)	0.001	--	--	0.48 (0.02, 0.94)	0.04
*C. leptum* group, copies*10^8^/g	-0.02 (-0.67, 0.62)	0.94	--	--	--	--
*Bacteroides*, copies*10^8^/g	-0.10 (-0.60, 0.41)	0.7	--	--	--	--
*Bifidobacterium*, copies*10^6^/g	0.14 (-0.09, 0.38)	0.23	--	--	--	--
*A. muciniphila*, copies*10^5^/g	-0.14 (-0.30, 0.02)	0.08	--	--	--	--
*E. coli*, copies*10^8^/g	-0.14 (-0.48, 0.20)	0.42	--	--	--	--
*F. prausnitzii*, copies*10^7^/g	0.09 (-0.20, 0.38)	0.52	--	--	--	--
**Laboratory data**						
Log-formed HbA1c	-0.10 (-0.31, 3.00)	0.95	--	--	--	--
Log-formed creatinine	3.49 (1.53, 5.44)	0.001	--	--	--	--
Hemoglobin, g/dl	0.47 (0.29, 0.66)	<0.001	--	--	--	--
Log-formed albumin	9.83 (0.94, 18.71)	0.03	--	--	--	--
Calcium, mg/dl	0.90 (-1.80, 0.01)	0.05	--	--	--	--
Log-formed phosphate	-5.26 (-9.96, -0.57)	0.03	--	--	--	--
Uric acid, mg/dl	0.08 (-0.15, 0.31)	0.49	--	--	--	--
Log-formed cholesterol	-3.18 (-6.63, 0.28)	0.07	--	--	--	--
Log-formed triglyceride	0.80 (-0.60, 2.19)	0.26	--	--	--	--
