# Supplementary material for: The Association of Targeted Gut Microbiota with Body Composition in Type 2 Diabetes Mellitus
Source: Int J Med Sci. 2021 Jan 1;18(2):511–9. doi: 10.7150/ijms.51164 (PMC7757146; doi:10.7150/ijms.51164)
Supplement: Supplementary file 1 — Supplementary figures and tables. [file ijmsv18p0511s1.pdf]

Supplementary Table 1. The primers used in real-time quantitative PCR.

Target	Primer (5'-3')	References
Phylum: *Firmicutes*	F: GGAGYATGTGGTTTAATTCGAAGCA	[1]
	R: AGCTGACGACAACCATGCAC	
C. leptum group (IV)	F: GTTGACAAAACGGAGGAAGG	[2]
	R: GACGGGCGGTGTGTACAA	
F. prausnitzii	F: AGATGGCCTCGCGTCCGA	[2]
	R: CCGAAGACCTTCTTCCTCC	
Phylum: *Bacteroidetes*	F: GGARCATGTGGTTTAATTCGATGAT	[1]
	R: AGCTGACGACAACCATGCAG	
Bacteroides	F: GTCAGTTGTGAAAGTTTGC	[2]
	R: CAATCGGGAGTTCTTCGTG	
Phylum: *Actinobacteria*		
Bifidobacterium	F: AGGGTTCGATTCTGCTCAG	[2]
	R: CATCCGGCATTACCACCC	
Phylum: *Proteobacteria*		
E. coli	F: CATGCCGCGTGTATGAAGAA	[3]
	R: CGGGTAACGTCAATGAGCAAA	
Phylum:*Verrucomicrobia*		
A. muciniphila	F:CAGCACGTGAAGGTGGGGAC	[4]
	R:CCTTGCGGTTGGCTTCAGAT	

References

1. Guo X, Xia X, Tang R, Wang K. Real-time PCR quantification of the predominant bacterial divisions in the distal gut of Meishan and Landrace pigs. Anaerobe. 2008; 14: 224-8.
2. Wang W, Chen L, Zhou R, Wang X, Song L, Huang S, et al. Increased proportions of *Bifidobacterium* and the *Lactobacillus* group and loss of butyrate-producing bacteria in inflammatory bowel disease. J Clin Microbiol. 2014; 52: 398-406.
3. Penders J, Thijs C, Vink C, Stelma FF, Snijders B, Kummeling I, et al. Factors influencing the composition of the intestinal microbiota in early infancy. Pediatrics. 2006; 118: 511-21.
4. Karlsson CL, Onnerfalt J, Xu J, Molin G, Ahrne S, Thorngren-Jerneck K. The microbiota of the gut in preschool children with normal and excessive body weight. Obesity (Silver Spring). 2012; 20: 2257-61.

Supplemental Table 2. The microbiota of the study subjects.

	LTI≦median N=91	LTI>median N=88	*P* value
Firmicutes, copies*10^9/g	4.18 (2.25, 7.73)	6.13 (2.89, 10.36)	0.02
Bacteroidetes, copies*10^9/g	10.71 (4.70, 24.64)	9.27 (4.09, 17.81)	0.23
Phyla F/B ratio	0.42 (0.20, 0.86)	0.64 (0.35, 1.52)	0.01
C. leptum group*10^8/g	6.6 (2.88, 12.53)	8.05(2.62, 17.82)	0.52
Bacteroides, copies*10^9/g	2.14 (1.00, 5.22)	1.98 (0.88, 3.66)	0.36
Bifidobacterium, copies*10^6/g	1.77 (0.69, 11.52)	1.81 (0.36, 10.16)	0.42
A. muciniphila, copies*10^5/g	0.17 (0.05, 317.92)	0.13 (0.04, 86.53)	0.44
E. coli, copies*10^8/g	1.18 (0.35, 7.38)	1.23 (0.31, 4.85)	0.63
F. prausnitzii, copies*10^7/g	9.35 (1.95, 26.38)	11.62 (2.47, 35.70)	0.53

LTI, lean tissue index.

Supplementary Table 3. The determinants of fat tissue index in type 2 diabetic patients.

	Univariate	
	β (95%Cl)	*p* value
**Microbiome**		
Log-formed *Firmicutes*	-0.17(-3.05,0.11)	0.07
Log-formed *Bacteroidetes*	-0.34(-1.31,0.64)	0.50
Log-formed *Firmicutes/Bacteroidetes* ratio	-0.51(-1.78,0.77)	0.43
Log-formed *C. leptum* group	-0.43(-1.68,0.82)	0.50
Log-formed *Bacteroides*	-0.49(-1.46,0.49)	0.33
Log-formed *Bifidobacterium*	-0.18(-0.64,0.27)	0.43
Log-formed *A. muciniphila*	-0.11(-0.42,0.21)	0.51
Log-formed *E. coli*	-0.48(-1.14,0.18)	0.16
Log-formed *F. prausnitzii*	0.02(-0.55,0.58)	0.96

Supplementary Table 4. The microbiota of the study subjects stratified by metformin usage.

	Metformin usage N=146	Non-metformin usage N=33	*p* value
Firmicutes, copies x 10^9/g	5.37 (2.56,8.40)	4.38 (1.88,10.13)	0.78
Bacteroidetes, copies x 10^9/g	10.48 (4.40,19.87)	7.16 (4.13,23.99)	0.53
Phyla F/B ratio	0.53 (0.24,1.21)	0.61 (0.24,0.99)	0.91
C. leptum group x 10^8/g	7.65 (2.63,13.32)	8.33 (2.47,18.41)	0.66
Bacteroides, copies x 10^9/g	2.19 (0.93,4.35)	1.50 (0.88,3.29)	0.42
Bifidobacterium, copies x 10^6/g	1.63 (0.17,9.17)	3.29 (0.26,15.21)	0.25
A. muciniphila, copies x 10^5/g	0.14 (0.05,167.50)	0.15 (0.05,623.94)	0.70
E. coli, copies x 10^8/g	1.23 (0.39,6.69)	1.17 (0.15,4.36)	0.13
F. prausnitzii, copies x 10^7/g	10.80 (1.93,31.64)	9.34 (4.50,26.37)	0.86
